# Reply to “Comment on the ‘Ground Water Chemistry Changes before Major Earthquakes and Possible Effects on Animals’, by R. A. Grant, T. Halliday, W. P. Balderer, F. Leuenberger, M. Newcomer, G. Cyr and F. T. Freund. *Int. J. Environ. Res. Public Health*, 2011, *8*, 1936–1956” from Friedemann Freund, Rachel Grant and Co-Authors

**DOI:** 10.3390/ijerph9072343

**Published:** 2012-07-02

**Authors:** Friedemann Freund, Rachel A. Grant

**Affiliations:** 1 Ames Research Center, National Aeronautics and Space Administration (NASA), Earth Science Division, Code SGE, Moffett Field, CA 94035, USA; 2 Department of Life Sciences, Anglia Ruskin University, East Road, Cambridge CB1 1PT, UK; Email: rachel.grant@anglia.ac.uk

We fully agree with Dr. Vassiliki Katsika-Tsigourakou that there is more than one possible explanation for the wide range of electromagnetic (EM) field bioeffects reported in the literature. In order to generate EM fields electric currents need to flow that oscillate. Currents that flow through the ground also generate electrical potentials. Such potentials can lead to electrochemical reactions at ground-water interfaces such as the demonstrated oxidation of water to hydrogen peroxide [1]. EM emissions and electrochemical reactions are therefore manifestations of the same physical process in the natural environment.

Unusual animal behavior before major earthquakes has been reported for centuries. With few exceptions in recent years, when systematic studies of animal behavior were fortuitously underway at the time of a nearby major earthquake, all observations fall under the rubric “anecdotal”. One may argue that every discovery starts with a serendipitous observation, made haphazardly “out of the blue”. To reach a deeper understanding of the underlying causes requires a large investment in time and financial resources. This is particularly true for “rare” events such as those linked to major earthquakes. 

For our report on the unusual behavior of toads we had the benefit of such a rare coincidence: an ongoing behavioral study of toads in a lake in Italy and the nearby L’Aquila earthquake of 6 April 2009 [2]. In our interpretation we focused on the possibility of electrochemical reactions driven by the flow of stress-activated electronic charge carriers through the Earth’s crust and their entry into the body of water. The same process—currents flowing through the ground—will produce EM emissions. If these EM emissions fall into the extremely low frequency (ELF) range around 1 Hz, they will be felt both in the water and on land. If the EM frequencies fall into the kHz range, the waves will be strongly absorbed by the water. In this case water would provide protection from the effects of such EM waves. 

The observation was that, prior to the earthquake, the toads left the lake in the midst of their breeding season. The toads disappeared not only from the lake but also from the wet lowlands, suggesting that they sought refuge on dry land. This selective behavior is consistent with an aversive stimulus felt in the water and on moist ground, but not on dry land. This is why we focussed our discussion on an electrochemical reaction at the ground-water interface, which causes water to be oxidized to hydrogen peroxide and may cause additional, biologically relevant oxidation reactions. By contrast, EM emissions are unlikely to be able to elicit specific hydrophobic behavior in these amphibians. However, we agree with Dr. Vassiliki Katsika-Tsigourakou that EM emissions and other pre-earthquake processes cannot be ruled out. 
